# Simon’s Manual of Chemistry

**Published:** 1902-01

**Authors:** 


### Bibliography.

Simon's Manual of Chemistry. A Guide to Lectures and Laboratory Work for Beginners in Chemistry, specially adapted for Students of Medicine, Pharmacy, and Dentistry. By W. Simon, Ph.D., M.D., Professor of Chemistry in the College of Physicians and Surgeons of Baltimore, in the Maryland College of Pharmacy, and in the Baltimore College of Dental Surgery. Seventh Edition. Thoroughly revised and much enlarged. In one octavo volume of six hundred and thirteen pages, with sixty-six engravings, one colored spectra plate, and eight colored plates representing sixty-four of the most important chemical reactions. Cloth, $3.00, net. Lea Brothers & Co., Publishers, Philadelphia and New York, 1901.

The seventh edition of a book means an appreciation that entirely settles its value and inclines the reviewer to hesitate to express an adverse opinion if such be felt, but in the case of the present volume the constant demand is fully justified by its contents and the methods adopted in its preparation.

The author has the happy faculty of combining instruction and, at the same time, holding the interest of the student, and this he accomplishes by a singular lucidity of expression with condensation of subject matter. The elementary principles of chemistry, generally so difficult for the beginner to master, are not only made clear, but are impressively forced upon the reasoning intelligence, and are silently made part of the mental capital of the student without perceptible effort. This is the gift of the true teacher, and Professor Simon seems to possess it in a remarkable degree.

The author in his preface to this edition says of it, "The call for a new—the seventh—edition of this manual has afforded the author an opportunity to incorporate, as far as practicable, the many important and latest results of scientific progress. At the same time he has complied with the request of many teachers to present more fully than was done heretofore the parts on *chemical physics* and on *physiological chemistry.*

"It is hoped that with these alterations and additions the manual will fully accomplish its object,—viz., *to furnish to the student in concise form a clear presentation of the science, an intelligent discussion of those substances which are of interest to him, and a reliable guide to his work in the laboratory.*"

The student will find this statement not too strongly written, for it is rare to find a book of this size more free from error.

The attention of the author is called to one of these on page 130, in the paragraph devoted to nitrogen monoxide, where the discoverer of anæsthesia is given as Dr. *Howard* Wells. It should have been printed *Horace.*

In the valuable chapter on Physiological Chemistry, in considering the tissues of the teeth, the author uses the term *fangs* as applied to the roots of teeth. This has been obsolete in dentistry for many years.

The illustrations are superior, especially the colored spectra plate and the eight colored plates representing sixty-four chemical reactions.

This work should have an important place in dental colleges among the series of text-books, for in most respects it is better adapted for this purpose than many of those at present in use.

The book has been published with the usual care of the well-known house of Lea Brothers & Co., nothing being omitted to make it worthy the subject and the author.

